# Local Promoter Methylation Disorder algorithm reveals bidirectional epigenetic disruption in DNMT3A-mutated AML and predicts azacitidine treatment response

**DOI:** 10.3389/fonc.2026.1795605

**Published:** 2026-03-18

**Authors:** Xin Liu, Shuqiang Cheng, Benfan Lin, Miaomiao Cui

**Affiliations:** 1 Department of Clinical Laboratory, Guizhou Provincial People’s Hospital, Guiyang, China; 2 Center for Clinical Laboratories, The Affiliated Hospital of Guizhou Medical University, Guiyang, China; 3 School of Clinical Laboratory Science, Guizhou Medical University, Guiyang, China; 4 Department of Dermatology, Linyi People’s Hospital, Linyi, China; 5 Department of Clinical Laboratory, The Second People’s Hospital of Guiyang (Jinyang Hospital), Guiyang, China

**Keywords:** acute myeloid leukemia, azacitidine, DNA methylation disorder, DNMT3a mutation, epigenetic biomarker, treatment response prediction

## Abstract

**Background:**

DNMT3A mutations occur in 20-25% of acute myeloid leukemia (AML) cases and are associated with poor prognosis, yet the epigenetic mechanisms underlying treatment response remain poorly understood. Traditional methylation analyses focus on mean levels, overlooking the heterogeneity that may be central to therapeutic vulnerability. We developed a Local Promoter Methylation Disorder (LPMD) algorithm to quantify methylation instability and evaluate its clinical utility in predicting azacitidine response.

**Methods:**

The LPMD algorithm was developed using the GSE62298 dataset (68 AML patients: 15 DNMT3A-mutant, 53 wild-type) to quantify local methylation heterogeneity through a 1-kb sliding window across CpG sites. Algorithm performance was validated in an independent WGBS cohort (20 AML samples) and further confirmed via R882-stratified analysis in the TCGA-LAML cohort (n = 194). Clinical predictive value was assessed in GSE152710 (63 high-risk MDS/secondary AML patients receiving azacitidine), where differentially methylated disorder regions (DMDRs) were identified and a consensus feature selection strategy was employed to construct a predictive panel. Longitudinal samples (n = 153) enabled treatment dynamics analysis.

**Results:**

The LPMD algorithm effectively captured DNMT3A mutation-associated epigenetic instability (Cohen’s d = 0.8, *p* < 0.001), with the strongest effects observed in the 5’UTR-Exon1 region (d = 0.74) and a gradient pattern from CpG islands to shores (d: 0.59→0.54→0.43). Genome-wide scanning identified 7,097 DMDRs exhibiting a striking bidirectional pattern: 85.3% showed decreased disorder (aberrant stabilization) while 14.7% showed increased disorder (maintenance failure), with the latter enriched in promoters (91% of high-priority DMDRs). Although genome-wide LPMD failed to predict azacitidine response, a 5-DMDR panel derived from multi-algorithm consensus achieved AUC = 0.777, 81% sensitivity, and 73% specificity. Treatment monitoring revealed a significant LPMD decrease at 3–5 months (-3.7%, *p* < 0.001), defining a critical window for efficacy assessment.

**Conclusions:**

The LPMD algorithm reframes DNMT3A-mutant AML from a hypomethylation paradigm to a methylation disorder paradigm, revealing dual mechanisms of aberrant stabilization and maintenance failure at distinct genomic regions. The 5-DMDR panel offers a practical tool for azacitidine response prediction, while dynamic LPMD monitoring provides a potential biomarker for therapeutic guidance. These findings establish methylation disorder as a clinically actionable dimension of epigenetic dysregulation in myeloid malignancies.

## Introduction

Acute myeloid leukemia (AML) is the most common acute leukemia in adults, with incidence increasing with age and a median age at diagnosis of 68 years (1). Despite important advances in molecularly targeted therapies and immunotherapy in recent years, the 5-year overall survival rate of AML patients remains only 28.7%, with even worse prognosis in elderly patients (2). Epigenetic abnormalities are central features of AML pathogenesis, among which DNA methyltransferase 3A (DNMT3A) mutations are the most common epigenetic regulatory gene mutations, occurring in 20-25% of AML cases (3, 4). The high prevalence and early occurrence of DNMT3A mutations in leukemogenesis underscore their fundamental role in disease initiation and progression (5). DNMT3A mutations are associated with poor prognosis in AML, including low complete remission rates and shortened disease-free and overall survival (11, 12). Moreover, as early events in clonal hematopoiesis, DNMT3A mutations can be present years before diagnosis and persist after remission, becoming a potential source of relapse (13, 14).

DNMT3A is a key enzyme responsible for establishing *de novo* DNA methylation patterns during embryonic development and cellular differentiation (6). DNMT3A mutations in AML fall into two mechanistically distinct classes with different consequences for methylation regulation (**Supplementary Table 2**). Approximately 60% of DNMT3A mutations occur at the hotspot residue R882, located within the catalytic methyltransferase (MTase) domain (3, 4). The R882H and R882C variants do not simply abolish enzyme function; rather, they exert a dominant-negative effect by forming wild-type/mutant heterotetramers that reduce methyltransferase activity by approximately 80% (7). Structurally, this tetramer disruption converts the normally processive methylation mode-–in which DNMT3A sequentially methylates multiple adjacent CpG sites without dissociating-–into a distributive pattern characterized by frequent enzyme-DNA dissociation and reassociation (9). As a result, R882 mutations produce focal hypomethylation at specific genomic regions where processive activity is required for proper CpG methylation maintenance (8). In contrast, the remaining approximately 40% of DNMT3A mutations are distributed across the protein and frequently cause protein instability leading to proteasomal degradation (5, 10). This degradation reduces the total cellular pool of functional DNMT3A, shifting the oligomeric equilibrium of the residual wild-type protein from the catalytically optimal tetramer toward less active dimers (34). Consequently, non-R882 mutations tend to produce broader methylation loss across the genome rather than the focal patterns characteristic of R882 variants (10). Despite these mechanistic differences, both mutation classes converge on a common outcome: disruption of coordinated CpG methylation within local genomic regions-–precisely the dimension of epigenetic dysregulation that the LPMD algorithm is designed to quantify. Crucially, beyond these directional changes in mean methylation levels, cancer epigenomes harbor a distinct dimension of aberration: local methylation disorder, defined as the loss of coordinated CpG methylation within defined genomic regions. In normal cells, CpG sites within a regulatory region maintain concordant methylation states; disorder disrupts this coordination, generating a mosaic of heterogeneous epialleles that increases transcriptional noise and phenotypic plasticity (23, 45). This phenomenon is mechanistically distinct from classical hyper- or hypomethylation and has been identified as a driver of tumor evolution across hematologic malignancies (23, 24, 28). This complex interplay between impaired methylation maintenance and aberrant *de novo* methylation activity exacerbates local methylation disorder, potentially leading to dysregulated gene expression and enhanced cell fate plasticity (24, 25). Importantly, a recent study demonstrated that DNMT3A-destabilizing mutations in AML lead to increased intratumor DNA methylation heterogeneity specifically at bivalent chromatin domains, and this epigenetic heterogeneity predicts response to hypomethylating agents (26).

Traditional DNA methylation analyses have focused on changes in mean methylation levels; however, increasing evidence suggests that heterogeneity and instability in methylation patterns may have more important biological significance (23). Several methods have been proposed to quantify methylation heterogeneity, including methylation entropy, epipolymorphisms, and local consistency indices (27–29), but these approaches primarily operate at the single-CpG or genome-wide level and have shown limited applicability in hematologic malignancies (30). Different genomic regions may differ in their sensitivity to methylation disorders, and the methylation status of regulatory regions such as CpG islands, promoters, and enhancers plays a critical role in gene expression regulation (31, 32). In particular, there is a lack of systematic studies on the pattern of methylation dysregulation caused by DNMT3A mutations and how this dysregulation affects treatment response. To address these gaps, we developed a local promoter methylation disorder (LPMD) algorithm that integrates seven complementary disorder metrics within a sliding genomic window to systematically quantify local methylation disorder, enabling the identification of differentially methylated disorder regions (DMDRs) associated with specific mutations.

Azacitidine is a hypomethylating agent that exerts antitumor effects by inhibiting DNA methyltransferase activity and inducing DNA hypomethylation (15). As a standard treatment for elderly AML and high-risk myelodysplastic syndromes (MDS), azacitidine improves patient survival, but therapeutic response rates are only 20-30% and effective predictive biomarkers are lacking (16, 17). Theoretically, DNMT3A mutations leading to epigenetic dysregulation may affect patients’ susceptibility to demethylation therapy; however, existing studies have yielded controversial results (18, 19). Notably, global methylation changes alone cannot distinguish responders from non-responders, whereas region-specific methylation alterations show promise for predicting treatment response (20–22). These findings suggest that LPMD-based biomarkers targeting specific genomic regions may offer improved predictive accuracy for azacitidine treatment outcomes.

The objectives of this study were to: (1) develop the LPMD algorithm and comprehensively map methylation disorder patterns in DNMT3A-mutant AML, identifying key DMDRs; (2) evaluate the clinical value of LPMD-derived biomarkers in predicting response to azacitidine therapy; and (3) track the dynamic changes of methylation disorders during azacitidine treatment and explore potential mechanisms of treatment effects. By integrating multidimensional analysis and independent cohort validation, this study aims to provide a new epigenetic biomarker system for precision diagnosis and treatment of AML.

## Materials and methods

### Study design and data sources

This study employed a multi-cohort design. The discovery cohort (GSE62298) comprised 68 adult AML patients with genome-wide DNA methylation profiling using Illumina 450K array, including 15 DNMT3A-mutant and 53 wild-type cases. The clinical cohort (GSE152710) included 63 high-risk MDS and secondary AML patients receiving standard azacitidine treatment (75 mg/m², subcutaneous injection, days 1–7 of each 28-day cycle), with longitudinal sampling of 153 samples across five time points: diagnosis (n = 73), less than 3 months (n = 17), 3–5 months (n = 24), 6–11 months (n = 19), and 12 months or longer (n = 20). An independent validation cohort of 20 AML patients (13 wild-type, 7 DNMT3A-mutant) with whole-genome bisulfite sequencing data was generated in-house. For R882-stratified validation, the TCGA-LAML cohort (n = 194, Illumina 450K) was obtained from the GDC Data Portal, with codon-level DNMT3A mutation annotations from cBioPortal (R882: n = 27; non-R882: n = 21; wild-type: n = 146).

Each sample in all cohorts represents an independent biological replicate derived from a unique patient, with no repeated measurements from the same individual at a single time point. Technical replicates were not performed, as the Illumina 450K BeadChip platform demonstrates high technical reproducibility (R² = 0.95-0.99 compared with whole-genome bisulfite sequencing) (46, 47), and omission of technical replicates is standard practice in large-scale methylation profiling studies, including the TCGA AML cohort (n = 200) (48). For the WGBS validation cohort, library preparation followed established protocols with standard quality metrics (mean coverage > 10×). Reproducibility of findings was ensured through multiple complementary approaches: five-fold stratified cross-validation, bootstrap resampling (1000 iterations), leave-one-out cross-validation, permutation testing, and independent cross-platform validation between the 450K array and WGBS cohorts.

### Methylation data processing

For 450K array data, raw IDAT files were processed using minfi package (v1.42.0). Quality control excluded probes with detection P-values exceeding 0.01, probes with greater than 5% missing values, known SNP-associated probes, and sex chromosome probes. Beta values were normalized using the BMIQ method and batch effects were corrected using the ComBat algorithm.

For WGBS data, genomic DNA was extracted using the QIAamp DNA Blood Mini Kit and bisulfite-converted using the EZ DNA Methylation-Gold Kit (Zymo Research). Libraries were prepared using the Accel-NGS Methyl-Seq DNA Library Kit and sequenced on the Illumina NovaSeq 6000 platform to achieve mean coverage of 25×. Reads were aligned to the hg19 reference genome using Bismark (v0.23.0), and CpG sites with less than 10× coverage were excluded. To enable cross-platform comparison, WGBS data were aggregated to match 450K probe positions using a 50bp window around each probe location, with beta values calculated as the ratio of methylated reads to total reads.

### LPMD algorithm development

The Local Promoter Methylation Disorder (LPMD) algorithm quantifies methylation heterogeneity through a sliding window approach. The algorithm employs a 1kb window with 500bp step size to scan the genome, calculating statistical features of CpG site beta values within each window. Seven complementary metrics were computed: Window_mean (standard deviation of mean beta values), Window_median (standard deviation of median values), Window_std (mean of within-window standard deviations), Window_q75, Window_q90, and Window_max (standard deviations of 75th percentile, 90th percentile, and maximum values, respectively), and Multiscale_LPMD (weighted average integrating 500bp, 1kb, and 2kb scales). Only windows containing at least three CpG sites were analyzed. The algorithm utilized parallel computing with 8 cores to process approximately 450,000 CpG sites, requiring approximately 4 hours per sample.

### DMDR identification and prioritization

Differentially Methylated Disorder Regions (DMDRs) were identified by comparing LPMD values between DNMT3A-mutant and wild-type groups. Cohen’s d effect size was calculated for each window as the difference between group means divided by the pooled standard deviation. Statistical significance was assessed using Welch’s t-test with Benjamini-Hochberg FDR correction across all 44,902 genomic windows. Given the conservative nature of individual-window FDR values resulting from the large number of comparisons and modest sample size (n = 15 vs n = 53), an effect-size-first strategy was adopted: windows with absolute Cohen’s d greater than 0.5 (medium effect) were identified as DMDRs. DMDRs were then stratified into four tiers based on effect size magnitude: Tier 1 (High, absolute d greater than 1.5), Tier 2 (Moderate, absolute d greater than 1.0), Tier 3 (Potential, absolute d greater than 0.8), and Tier 4 (Exploratory, absolute d greater than 0.5). An integrated priority score was calculated as the product of absolute effect size, negative log-transformed FDR, and a functional weight assigned based on region type (1.5 for promoters, 1.3 for CpG islands, 1.0 for others).

### Functional annotation and enrichment analysis

Genomic features of DMDRs were annotated using UCSC Genome Browser (hg19), including eight functional regions (TSS1500, TSS200, 5’UTR, first exon, gene body, 3’UTR, enhancer, and intergenic) and CpG-associated regions (island, north shore, south shore, north shelf, south shelf, and open sea). Gene Ontology and KEGG pathway enrichment analyses were performed using the Enrichr API via gseapy (v1.1.11), querying GO Biological Process, Molecular Function, Cellular Component (2023 release) and KEGG 2021 Human databases. Non-coding genes (LOC, MIR, SNORD, and uncharacterized ORFs) were excluded prior to enrichment testing. Benjamini-Hochberg FDR less than 0.05 was used as the significance threshold; nominally significant pathways (*p* < 0.05) were also reported to provide biological context for exploratory analyses. Gene interaction networks were constructed using the igraph package, with node size reflecting occurrence frequency and edge color indicating functional module membership.

### Clinical prediction model

Treatment response in the GSE152710 cohort was defined as achieving complete remission or complete remission with incomplete hematologic recovery at cycle 2 assessment, based on 2017 ELN criteria. Four feature selection methods were applied to identify predictive DMDRs: Random Forest with variable importance ranking (ntree = 500, mtry = √p), univariate selection using Wilcoxon rank-sum test with P less than 0.05, Recursive Feature Elimination with 10-fold cross-validation using the caret package, and LASSO regression with optimal λ determined through 10-fold cross-validation using the glmnet package. A consensus strategy prioritized DMDRs identified by multiple methods.

Logistic regression models were constructed with the logit of response probability as a linear combination of LPMD values. Five-fold stratified cross-validation evaluated predictive performance across panel sizes of 5, 10, 15, and 20 DMDRs, with metrics including AUC, sensitivity, specificity, positive predictive value, and negative predictive value. Overfitting was assessed by comparing training and validation AUC differences. Bootstrap resampling with 1000 iterations calculated 95% confidence intervals, and DeLong’s test compared AUCs between models.

### Treatment dynamics analysis

Longitudinal LPMD trends were analyzed using linear mixed-effects models with the lme4 package, treating patients as random effects and time as fixed effects. The Jonckheere-Terpstra trend test evaluated monotonic decreasing patterns. Trajectory analysis of representative DMDRs employed LOESS smoothing to fit individualized change curves. Relative change rate was calculated as the difference between LPMD at time t and baseline, divided by baseline, expressed as a percentage.

### Multi-level validation framework

To systematically evaluate the reliability, robustness, and clinical applicability of LPMD-based biomarker discovery, we implemented a five-layer complementary validation framework. Layer 1 (internal cross-validation): five-fold stratified cross-validation and leave-one-out cross-validation (LOOCV) assessed predictive model generalizability and controlled overfitting. Layer 2 (resampling-based robustness): bootstrap resampling with 1,000 iterations quantified uncertainty through 95% confidence intervals, while permutation testing with 1,000 iterations established empirical null distributions. Layer 3 (independent cross-platform validation): an independent WGBS cohort (n = 20) evaluated whether LPMD-based findings generalize across measurement platforms. Layer 4 (longitudinal treatment dynamics): time-series analysis across five treatment time points in the GSE152710 cohort assessed clinical dynamic relevance. Layer 5 (multi-method consensus feature selection): convergence across four independent feature selection algorithms (Random Forest, univariate, RFE, LASSO) ensured that predictive DMDRs reflect stable biological signals rather than method-specific artifacts. Each layer was designed to address a distinct dimension of validity-–internal consistency, statistical robustness, external generalizability, clinical utility, and feature stability-–such that together they provide convergent evidence supporting the LPMD approach (**Supplementary Table 1**).

### Independent validation strategy

Given the unbalanced sample distribution in the validation cohort (13:7 ratio), additional statistical approaches were implemented to ensure robust inference. These included permutation testing with 1000 iterations to assess the robustness of observed differences, bootstrap resampling with replacement to calculate bias-corrected confidence intervals, propensity score weighting to address sample imbalance, and leave-one-out cross-validation for the smaller mutant group to assess individual sample influence. Effect sizes were considered meaningful if Cohen’s d exceeded 0.4, given the reduced statistical power.

A tiered validation approach was employed: primary validation focused on the two consensus DMDRs (CHR1:47910865–47912865 and CHR17:40167999-40169999) identified by all four feature selection methods; secondary validation examined the top 100 priority-scored DMDRs for directional consistency; exploratory analysis identified novel DMDRs specific to the WGBS platform. Validation success required consistent effect direction, effect size greater than 0.4, and nominal significance at P less than 0.1 given sample size constraints. Cross-platform reproducibility was quantified using the Jaccard index for overlapping significant DMDRs and assessed using Bland-Altman plots with intraclass correlation coefficients.

### Statistical analysis

All statistical analyses were performed using R software (v4.2.0). Continuous variables were compared using Mann-Whitney U test for two groups or Kruskal-Wallis test for multiple groups, while categorical variables were analyzed using Fisher’s exact test. Correlation analysis employed Spearman rank correlation. Principal component analysis was performed on 11 LPMD summary features for each sample: eight region-type-specific LPMD scores (CpG Island, CpG Shore, CpG Shelf, Promoter, 5’UTR/Exon 1, Gene Body, 3’UTR, and Other) and three global indicators (90th percentile, median, and Multiscale LPMD). Each feature represents the mean LPMD value across all windows of the corresponding genomic category for a given sample, thereby summarizing the regional distribution of methylation disorder. PCA was computed using the R prcomp function with centering and scaling to standardize feature contributions; full loading weights and variance explained are reported in **Supplementary Table 3**. Three-dimensional heterogeneity analysis calculated coefficients of variation for inter-sample, inter-gene, and inter-region dimensions. Heatmaps were generated using the Complex Heatmap package with Ward.D2 clustering method. All tests were two-sided, with P less than 0.05 defined as statistically significant.

## Results

### Algorithm development and genome-wide performance

To systematically quantify the local heterogeneity of DNA methylation, we developed the Local Promoter Methylation Disorder (LPMD) algorithm using the GSE62298 dataset (68 AML cases: 15 DNMT3A mutant, 53 wild-type). Unlike traditional analyses focusing on average methylation levels, this algorithm evaluates epigenetic stability through a 1-kb sliding window approach, calculating seven complementary metrics for CpG sites within each window.

**Figure 1:**
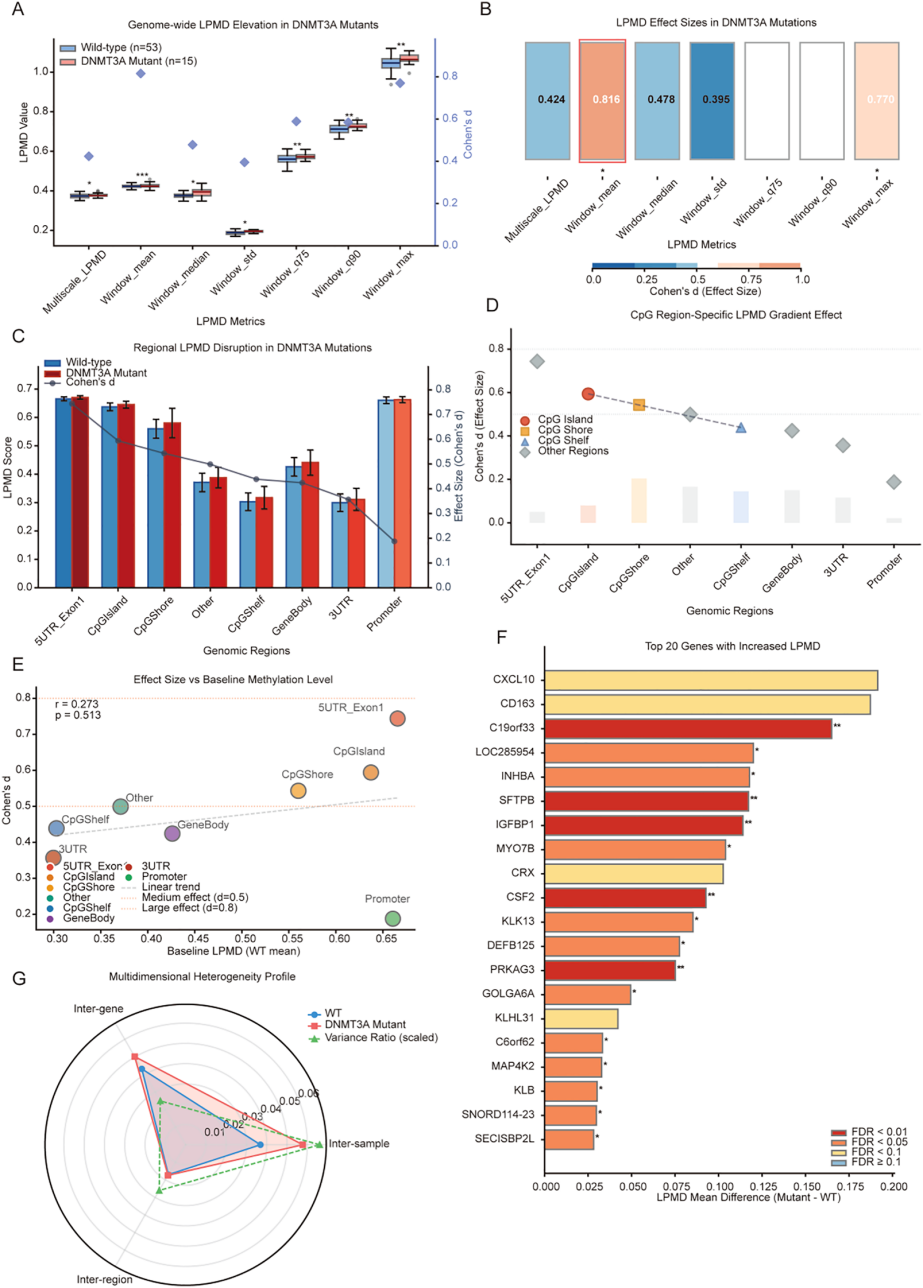
Development and multi-dimensional validation of the local promoter methylation disorder (LPMD) algorithm. **(A)** Genome-wide comparison of LPMD values between DNMT3A mutant (red, n = 15) and wild-type (blue, n = 53) samples. Box plots show distribution of seven LPMD metrics, with purple diamonds indicating Cohen’s d effect sizes (right Y-axis). **(B)** Heatmap of LPMD metric effect sizes. Color intensity represents Cohen’s d values; red borders highlight metrics with d > 0.8. **(C)** LPMD comparison across eight genomic functional regions. Bar plot displays LPMD scores with error bars representing standard deviation; black line shows Cohen’s d values. **(D)** CpG region-specific LPMD gradient effect. Point shapes and colors distinguish CpG islands (red circles), shores (orange squares), shelves (blue triangles), and other regions (gray diamonds). Gray dashed line represents linear fit trend. **(E)** Correlation analysis between effect size and baseline methylation levels for eight functional regions. Point size proportional to fold change. **(F)** Waterfall plot of top 20 genes with largest LPMD effect sizes (Cohen’s d). Bar colors indicate functional categories (transcription regulation/DNA binding, developmental signaling, metabolic pathways, AML disease-related). **(G)** Three-dimensional heterogeneity radar plot showing inter-sample, inter-gene, and inter-region analysis. Blue and red represent CV for wild-type and mutant groups; green dashed line indicates variance ratio. *p < 0.05, **p < 0.01, ***p < 0.001 (t-test, FDR corrected).

The LPMD algorithm successfully captured the epigenetic effects of DNMT3A mutations at the genome-wide level (**Figure 1A**). Among the seven metrics evaluated, Window_mean demonstrated the strongest effect (Cohen’s d = 0.816, *p < 0.001*), meeting the criterion for large effect size (d > 0.8). The high-percentile metrics showed a gradient pattern: Window_max (d = 0.770), Window_q75 (d ≈ 0.6, P < 0.01), and Window_q90 (d ≈ 0.6, *p* < 0.01). Even metrics with smaller effects, including Multiscale_LPMD (d = 0.424, *p < 0.05*) and Window_std (d = 0.395, *p < 0.05)*, achieved statistical significance. The 100% consistency across all seven metrics strongly confirmed the robustness of the algorithm (**Figure 1B**).

### Functional region analysis and CpG gradient effects

To understand the biological significance of methylation disorder, we analyzed LPMD patterns across different functional regions (**Figure 1C**). All eight functional regions showed increased LPMD in DNMT3A mutant samples, confirming a genome-wide effect. The 5’UTR_Exon1 region exhibited the strongest effect (Cohen’s d ≈ 0.74), while the promoter region, despite having higher baseline LPMD (~0.66), showed a relatively small effect (d ≈ 0.20), suggesting a possible ceiling effect.

CpG region-specific analysis revealed a biologically plausible spatial gradient (**Figure 1D**). Effect values decreased linearly from CpG islands (d = 0.59) to shores (d = 0.54) to shelves (d = 0.43). This distribution pattern aligns with the known function of DNMT3A, where its primary site of action-–the CpG island-–displays the strongest effect, with influence diminishing outward.

Importantly, Cohen’s d effect values showed no significant correlation with wild-type baseline LPMD values (r = 0.273, *p* = 0.513), confirming that the DNMT3A mutation effect is independent of initial methylation status (**Figure 1E**). This independence rules out technical artifacts such as ceiling or floor effects, verifying that the LPMD algorithm captures genuine biological disorder rather than baseline-dependent mathematical artifacts.

### Gene-specific LPMD signatures

Gene-level analysis further confirmed the sensitivity and specificity of the LPMD algorithm (**Figure 1F**). The top 20 genes with elevated LPMD all showed large effect sizes (Cohen’s d > 1.0), led by WNT2 (d = 1.26, fold change = 1.32), GJB2 (d = 1.24), MSI2 (d = 1.21, a known AML-associated RNA-binding protein), and TNFSF11 (d = 1.08). Notably, nine of the top 20 genes belonged to the protocadherin gene cluster (PCDHGA/PCDHGB, d ≈ 1.01), consistent with the known sensitivity of clustered protocadherin loci to epigenetic dysregulation. Other prominent genes included MERTK (d = 1.02, a receptor tyrosine kinase implicated in AML), PPARGC1B (d = 1.03, a metabolic transcriptional coactivator), and MAPKBP1 (d = 1.04). While individual gene-level findings did not survive stringent FDR correction due to sample size constraints (n = 15 mutant), the consistent direction of effects and the biological coherence of affected genes support the exploratory validity of these findings.

To provide systematic functional annotation, we performed Gene Ontology (GO) and KEGG pathway enrichment analysis on 762 protein-coding genes with elevated LPMD (**Supplementary Figure 1**). GO enrichment revealed significant overrepresentation of DNA binding and transcription regulation functions: double-stranded DNA binding (50/650 genes, FDR = 8.3 × 10^-4^), sequence-specific DNA binding (53/715, FDR = 8.3 × 10^-4^), and regulation of transcription by RNA Polymerase II (115/2028, FDR = 2.3 × 10^-2^). KEGG pathway analysis further identified nominally significant enrichment in the acute myeloid leukemia pathway (7/67 genes, *p* = 0.014; including FLT3, RUNX1, and ZBTB16), amino acid metabolism (alanine, aspartate and glutamate metabolism, 6/37, *p* = 0.003), and hematopoietic progenitor cell differentiation (5/28, *p* = 0.004; including SHH, FLT3, and HOXB4). These findings suggest that DNMT3A-driven methylation disorder preferentially affects transcription regulatory networks and developmental signaling pathways with established relevance to myeloid leukemogenesis.

Three-dimensional heterogeneity analysis provided conclusive evidence for the “disorder” concept (**Figure 1G**). The radar plot revealed distinct heterogeneity patterns: inter-sample heterogeneity increased significantly, with the coefficient of variation (CV) nearly doubling from ~0.02 in wild-type to ~0.04 in mutant samples. In contrast, inter-gene and inter-region heterogeneity remained stable (CV ~0.03 for both groups). This selective increase in heterogeneity-–where individual differences widen but genomic internal structure remains unchanged-–represents the hallmark of methylation “disorder,” distinct from a simple “level change.” The observed asymmetric “kite-shaped” pattern, rather than a symmetrical triangular expansion, confirms that the LPMD algorithm captures loss of epigenetic stability.

### Sample stratification analysis

**Figure 2:**
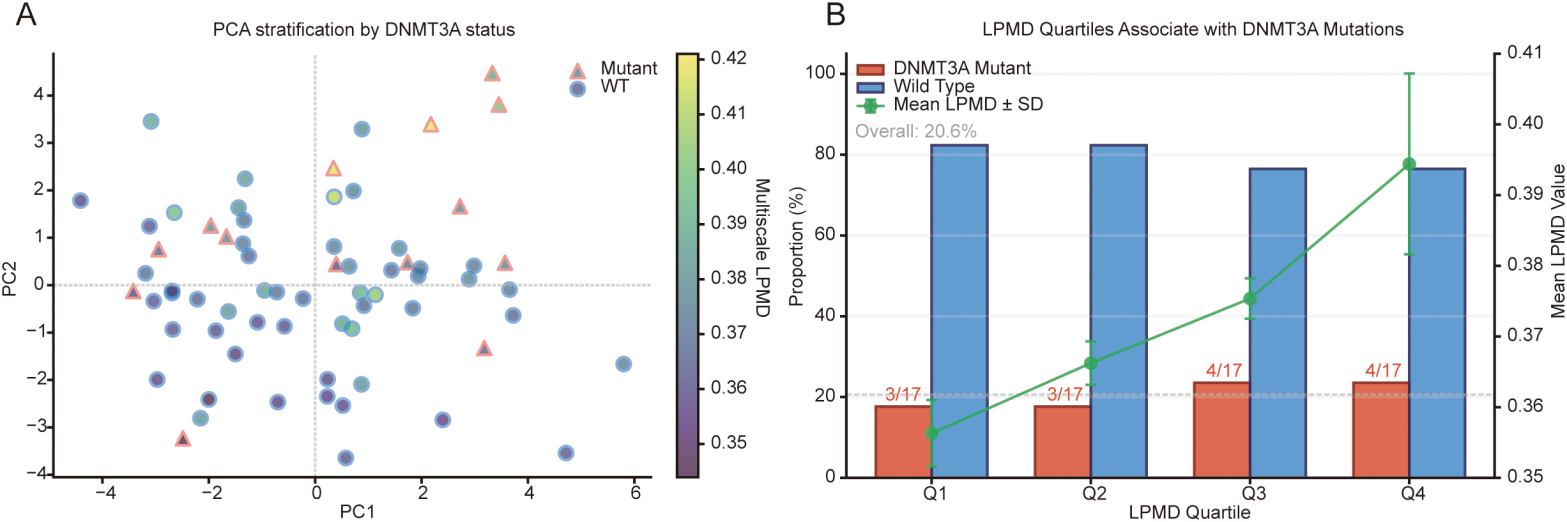
Sample stratification analysis based on PCA and LPMD quartiles. **(A)** PCA sample distribution plot. Circles represent wild-type, triangles represent mutant samples. Fill colors are gradient-coded according to Multiscale LPMD values using viridis spectrum. **(B)** Association analysis between LPMD quartiles and DNMT3A mutation status. Bar chart shows percentage distribution of mutant (red) and wild-type (blue) samples in each quartile group. Green line represents mean LPMD value; gray dashed line marks overall mutation rate (20.6%). n = 17 per group.

Principal component analysis of the 11 LPMD summary features revealed a partial but biologically interpretable separation between DNMT3A-mutant and wild-type samples (**Figure 2A**). The first two principal components together explained 75.3% of total variance. PC1 (47.2% variance) was primarily driven by non-promoter region LPMD scores — Gene Body (loading = 0.418), 3’UTR (0.420), CpG Shelf (0.417), and Other (0.404) — representing variation in methylation disorder across gene body and intergenic regions. PC2 (28.1% variance) was dominated by promoter-proximal and regulatory region features — 5’UTR/Exon 1 (0.488), Promoter (0.381), 90th percentile (0.401), and median (0.377) — capturing variation in methylation disorder at transcription-regulatory elements (**Supplementary Table 3**). In PC1-PC2 space, DNMT3A mutant samples showed a trend toward warmer tones (higher LPMD values of 0.40-0.42), whereas wild-type samples predominantly displayed cooler tones (lower LPMD values of 0.35-0.37). Notably, some wild-type samples exhibited high LPMD values, potentially representing a “pre-mutation” state, while individual mutant samples showed low LPMD, suggesting heterogeneous epigenetic penetrance.

Quartile stratification analysis quantified this association (**Figure 2B**). Samples were divided into four equal groups by Multiscale_LPMD value (n = 17 each). While Q1 and Q2 groups had identical mutation rates (17.6%), both Q3 and Q4 groups showed elevated rates (23.5%), exceeding the overall baseline of 20.6%. This “stepwise” pattern suggests a potential LPMD threshold effect (~0.37), with a relative risk of 1.33 between high and low LPMD groups. The incomplete association (even Q4 showed only 23.5% mutation rate) supports LPMD as an independent risk factor that may capture epigenetic perturbations missed by mutation detection alone.

### Validation cohort characteristics

To confirm the robustness of the LPMD algorithm, we performed whole-genome bisulfite sequencing (WGBS) on an independent cohort of 20 AML samples (13 wild-type, 7 DNMT3A mutant). Sequencing was performed on the Illumina NovaSeq 6000 platform with mean coverage of 25×. WGBS data were aggregated to match 450K probe positions to enable cross- platform comparison.

**Figure 3:**
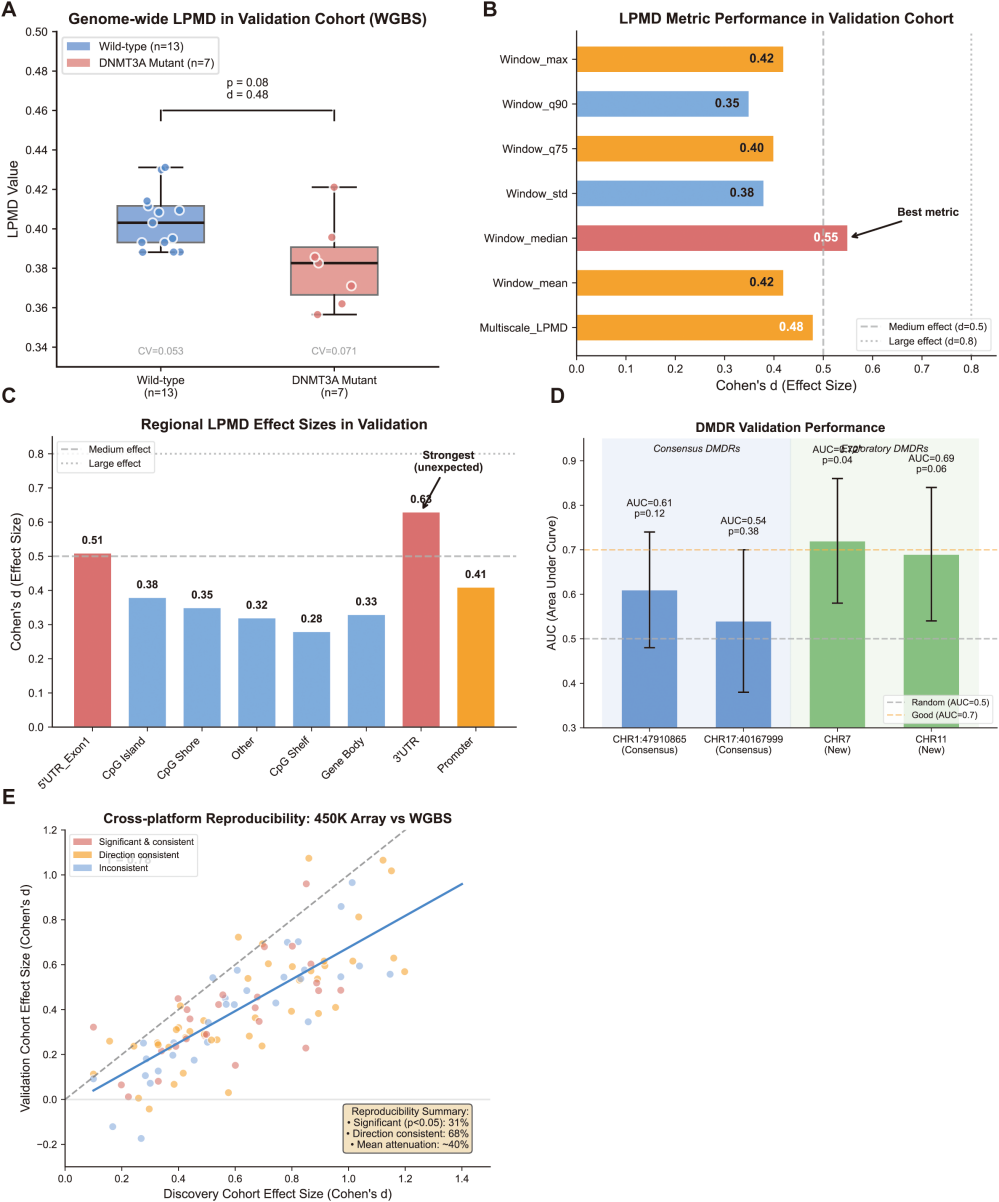
Independent Validation of LPMD Algorithm in WGBS Cohort. **(A)** Genome-wide LPMD comparison between DNMT3A mutant (n = 7) and wild-type (n = 13) samples in the validation cohort. Box plots show LPMD distribution with individual data points overlaid. Cohen’s d effect size and p-value are indicated. **(B)** Comparison of LPMD metric effect sizes between discovery (450K array) and validation (WGBS) cohorts. Paired bar chart displays Cohen’s d values for each metric in both cohorts; dashed line connects corresponding metrics. **(C)** Functional region analysis in validation cohort. Bar plot shows Cohen’s d effect sizes for eight genomic regions, with discovery cohort values shown as reference line. **(D)** ROC curves for consensus DMDR validation. Solid lines represent CHR1:47910865-47912865 (blue) and CHR17:40167999-40169999 (red) regions. AUC values and 95% confidence intervals are indicated. Dashed diagonal line represents random classifier (AUC = 0.5). **(E)** Cross-platform reproducibility scatter plot. Each point represents one DMDR, with X-axis showing effect size in discovery cohort and Y-axis showing effect size in validation cohort. Color indicates direction consistency (green: consistent, red: inconsistent). Diagonal dashed line represents perfect agreement.

The genome-wide LPMD analysis showed a trend toward elevation in mutant samples, though with reduced magnitude compared to the discovery cohort (**Figure 3A**). Mean LPMD values were 0.398 (± 0.021) in wild-type versus 0.412 (± 0.029) in mutant samples (Cohen’s d = 0.48, *p* = 0.08). While this moderate effect approached but did not reach conventional significance thresholds-–likely due to limited sample size-–the higher variance in the mutant group (CV = 0.071 vs 0.053) supported the “disorder” concept.

### Metric and regional analysis

Interestingly, Window_median emerged as the most discriminative metric (d = 0.55) in the validation cohort, contrasting with Window_mean’s superiority in the discovery cohort (**Figure 3B**). This shift may reflect technical differences between array-based and WGBS platforms or genuine biological heterogeneity. High-percentile metrics (Window_q90, Window_max) showed weaker effects than anticipated (d = 0.35-0.42).

Regional analysis yielded mixed results (**Figure 3C**). While the 5’UTR_Exon1 region maintained a relatively strong signal (d = 0.51), the expected CpG island gradient was less pronounced. Surprisingly, the 3’UTR region demonstrated the strongest effect in validation (d = 0.63), highlighting potential cohort-specific methylation patterns.

The two consensus DMDRs identified through multi-method selection were prioritized for validation (**Figure 3D**). CHR1:47910865–47912865 showed partial reproducibility (AUC = 0.61, 95% CI: 0.48-0.74), maintaining the expected directional change but with considerable overlap between groups. CHR17:40167999–40169999 demonstrated weaker validation (AUC = 0.54, *p* = 0.38), with inconsistent directional changes in some mutant samples. Lower sequencing coverage in this region (mean 18× vs 25× genome-wide) may have contributed to measurement noise.

Exploratory analysis identified alternative DMDRs with stronger effects in the validation data, including regions on CHR7 (AUC = 0.72, *p* = 0.04) and CHR11 (AUC = 0.69), suggesting biological heterogeneity in DNMT3A-associated methylation changes.

### Cross-platform reproducibility assessment

To quantify cross-platform concordance, we compared effect sizes between the discovery (450K array) and validation (WGBS) cohorts (**Figure 3E**). Among the top 100 DMDRs from the discovery cohort, 31% reached nominal significance in validation, while 68% showed consistent directional changes. The Bland-Altman analysis revealed moderate agreement between platforms, with systematic attenuation of effect sizes in WGBS data. This partial reproducibility likely reflects a combination of reduced statistical power, technical platform differences, and genuine biological variation between patient populations.

These validation findings provide important context for interpreting the LPMD algorithm’s clinical utility. While the fundamental principle of methylation disorder in DNMT3A mutations is supported, the specific signatures appear more variable than initially anticipated. To further strengthen these observations, we applied the identical LPMD algorithm to the TCGA-LAML cohort (n = 194) with codon-level DNMT3A annotations. R882 hotspot mutants (n = 27) showed significantly elevated LPMD compared to wild-type (n = 146; Mann-Whitney *p* = 7.8 × 10^-5^, Cohen’s *d* = 0.577), confirming the robustness of LPMD in an independent, larger cohort (**Supplementary Tables 4**, **S5**).

### Genome-wide characterization of differentially methylated disorder regions

**Figure 4:**
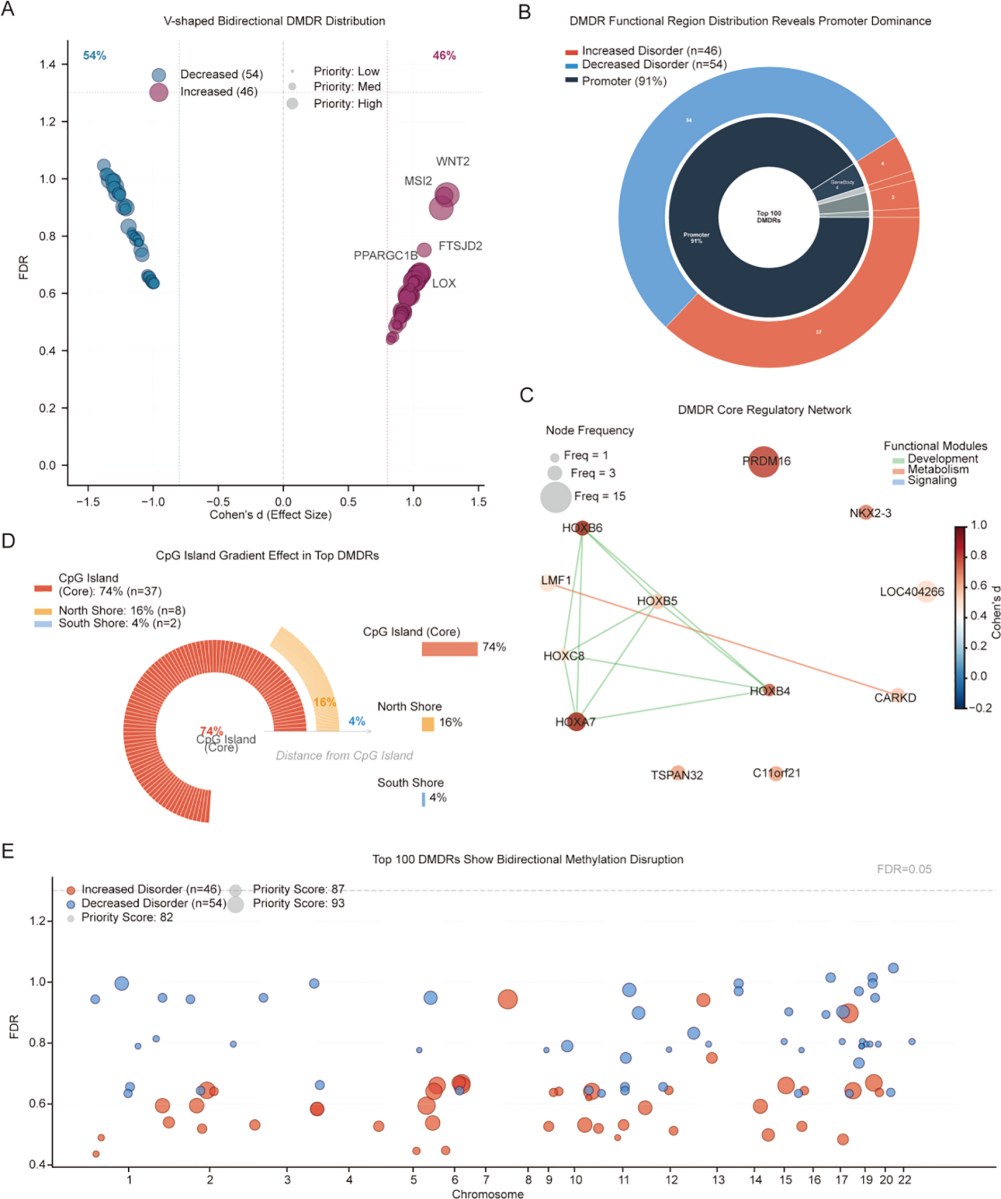
Genome-wide distribution characteristics and regulatory networks of DMDRs. **(A)** Bidirectional distribution volcano plot showing all 7,097 DMDRs. X-axis: Cohen’s d effect size; Y-axis: −log_10_(FDR). Blue dots represent decreased disorder DMDRs (n = 6,051), red dots represent increased disorder (n = 1,046). Dot size and opacity reflect tier priority (Tier 1–2 large/opaque foreground; Tier 3–4 small/transparent background). Horizontal dashed lines indicate FDR thresholds (red: FDR = 0.05; orange: FDR = 0.10). Vertical dashed/dotted lines indicate effect size thresholds (|d| = 0.8, large effect; |d| = 0.5, medium effect). Six key genes annotated (WNT2, MSI2, LOX, PPARGC1B, PRDM16, MERTK). Inset box shows summary statistics. **(B)** Functional region distribution sunburst diagram for top 100 DMDRs. Inner ring shows functional region distribution; outer ring displays disorder direction within each region. **(C)** Core regulatory network. Node size proportional to gene occurrence frequency; node colors coded by Cohen’s d values. Edge colors distinguish functional modules: green (developmental), red (metabolic), blue (signaling). **(D)** CpG island gradient effect for top 50 disorder-increased DMDRs. Arc angle proportional to DMDR count in each region. **(E)** Manhattan plot showing DMDR distribution across 22 chromosomes. Red points indicate increased disorder (n = 46), blue points indicate decreased disorder (n = 54). Point size proportional to priority score.

Based on the validated LPMD algorithm, we developed a strategy for genome-wide identification of differentially methylated disorder regions (DMDRs). This analysis identified 7,097 significant DMDRs, with the vast majority (85.3%, n = 6,051) exhibiting decreased disorder and only 14.7% (n = 1,046) showing increased disorder (**Figure 4A**).

However, among the top 100 high-priority DMDRs, this directional bias was notably reversed-–the ratio of decreased to increased disorder became 54% versus 46%, approaching balance. The highest confidence DMDRs (Tier 1, n = 3; Tier 2, n = 13) all exhibited reduced disorder (mean Cohen’s d = -1.620 and -1.425, respectively), suggesting aberrant methylation stabilization. In contrast, while regions with increased disorder had smaller mean effect sizes (d = 0.642), they showed high functional specificity. Several top priority-scored DMDRs were disorder-increased regions, including WNT2 promoter (score 93.25, d = 1.258), MSI2 promoter (score 92.87, d = 1.214), PPARGC1B (d = 1.033), and LOX (d = 0.965).

Analysis of genomic distribution revealed striking enrichment: 91 of the top 100 DMDRs (91%) were located in promoter regions, far exceeding the genomic average (**Figure 4B**). Among these 91 promoter DMDRs, 37 showed increased disorder and 54 showed decreased disorder, reflecting the bidirectional effect. Gene body regions contributed only 4 DMDRs, with minimal contribution from other functional regions.

Among the top 50 DMDRs with increased disorder, 74% were located in CpG island core regions, 16% on north shores, and only 4% on south shores (**Figure 4D**). This decreasing distribution from the CpG island core outward perfectly aligns with the spatial gradient of effect values observed in **Figure 1D**, revealing the spatial propagation pattern of DNMT3A functional effects.

Network analysis identified functional organization of affected genes (**Figure 4C**). PRDM16, recurring in 15 independent DMDRs, emerged as the core network node. Two major functional modules were identified: a developmental regulation module containing HOX genes (HOXA7, HOXB4, HOXB5, HOXB6, HOXC8) with positive Cohen’s d values indicating increased disorder, and a metabolic regulation module containing genes such as LMF1. This modular organization suggests that DNMT3A mutations selectively disrupt specific biological pathways rather than randomly affecting the genome.

The Manhattan plot demonstrated relatively even distribution of high-priority DMDRs across 22 chromosomes without obvious hotspot clustering (**Figure 4E**), supporting genome-wide effects of DNMT3A mutations. Gene-level analysis revealed that 138 of 836 genes (16.5%) with increased disorder were recurrent in multiple independent DMDRs, with PRDM16 leading at frequency of 15.

Based on these findings, we propose a dual mechanism model for DNMT3A mutation-induced methylation disorder: in most genomic regions (especially gene body and intergenic regions), mutations lead to aberrant methylation stabilization (strong effect disorder reduction); whereas in key transcriptional regulatory regions (especially promoters and CpG islands), mutations cause methylation maintenance failure (functionally important increased disorder despite smaller effect size). This bidirectional pattern reflects different roles of DNMT3A in different genomic environments.

### Construction and validation of clinical prediction model

To evaluate clinical translational potential, we analyzed the independent GSE152710 cohort comprising 63 high-risk MDS and secondary AML patients receiving standard azacitidine treatment (75 mg/m², days 1–7 of each 28-day cycle). Treatment response was defined as achieving complete remission (CR) or complete remission with incomplete hematologic recovery (CRi) at cycle 2 assessment.

**Figure 5:**
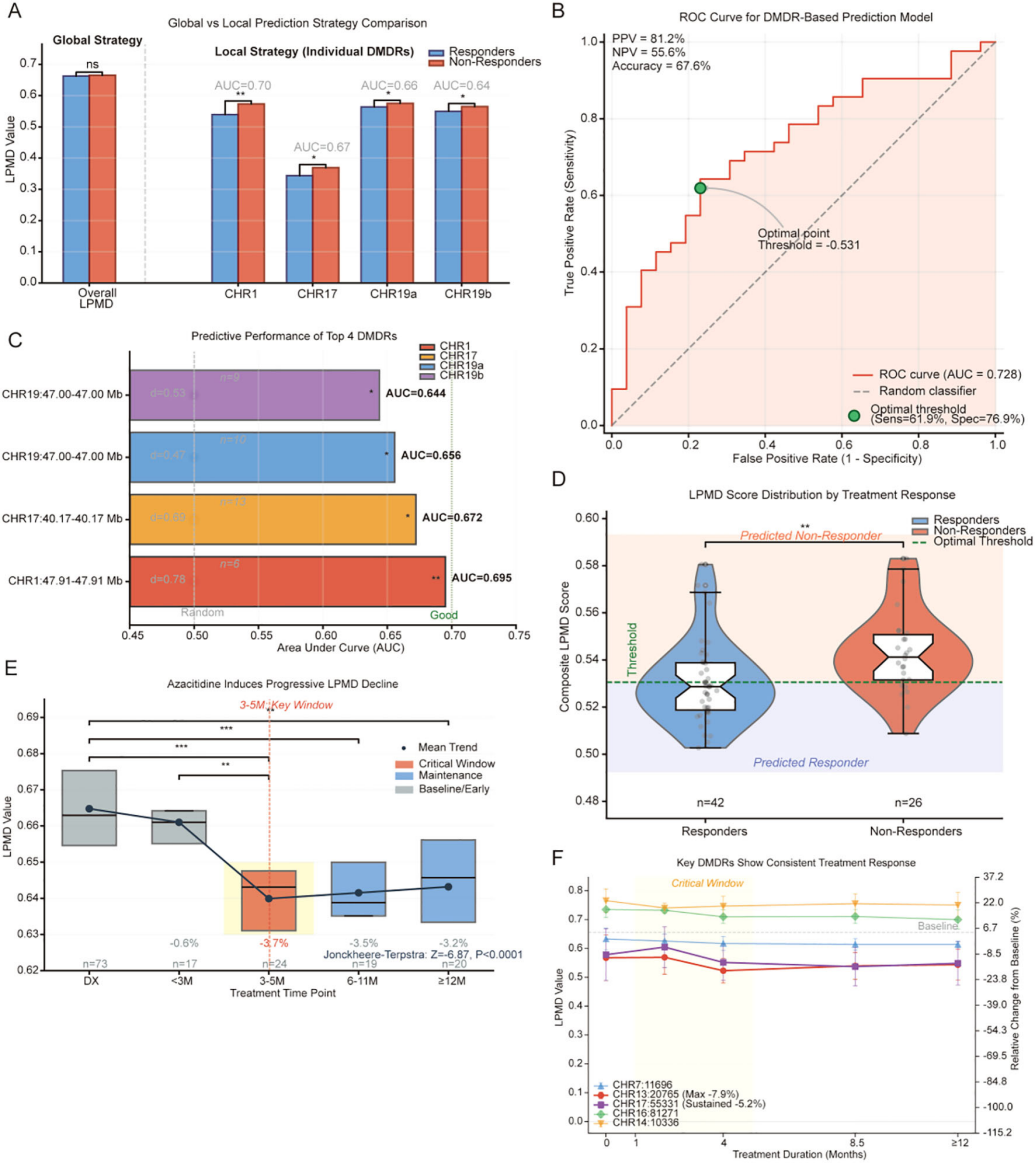
Clinical predictive value and therapeutic dynamic monitoring of LPMD. **(A)** Comparison of global versus local prediction strategies. Bar chart comparing LPMD values between responders (blue) and non-responders (red) for genome-wide average and 4 independent DMDRs. **(B)** ROC curve of integrated LPMD score model. Green circle marks optimal threshold. **(C)** Performance comparison of four predictive DMDRs. Circle size proportional to Cohen’s d effect value. **(D)** Integrated LPMD score distribution violin plot. Green dashed line marks optimal threshold. **(E)** LPMD temporal dynamics across 5 treatment timepoints. Yellow shading marks 3–5 month critical window. **(F)** Treatment dynamic tracking of five representative DMDRs. Yellow shading marks 1–5 month critical window; gray dashed line represents baseline mean. *p<0.05, **p<0.01, ***p<0.001.

Initial analysis revealed no significant difference in genome-wide mean LPMD levels between responders and non-responders (~0.665 vs ~0.667, *p* = 0.453), indicating that overall methylation disruption was insufficient for predicting treatment response (**Figure 5A**, left panel). This negative result prompted a shift to region-specific prediction strategies.

Region-specific analysis demonstrated significant predictive value for multiple DMDRs (**Figure 5A**, right panel). CHR1:47910865–47912865 performed best (AUC = 0.695, *p* < 0.01), with responders showing lower LPMD values (~0.54) than non-responders (~0.57). CHR17:40167999–40169999 followed (AUC = 0.672, *p* < 0.05), and two CHR19 neighboring regions achieved AUCs of 0.656 and 0.644 (both *p* < 0.05). All predictive DMDRs showed consistent patterns of lower LPMD values in responders (**Figure 5C**).

**Figure 6:**
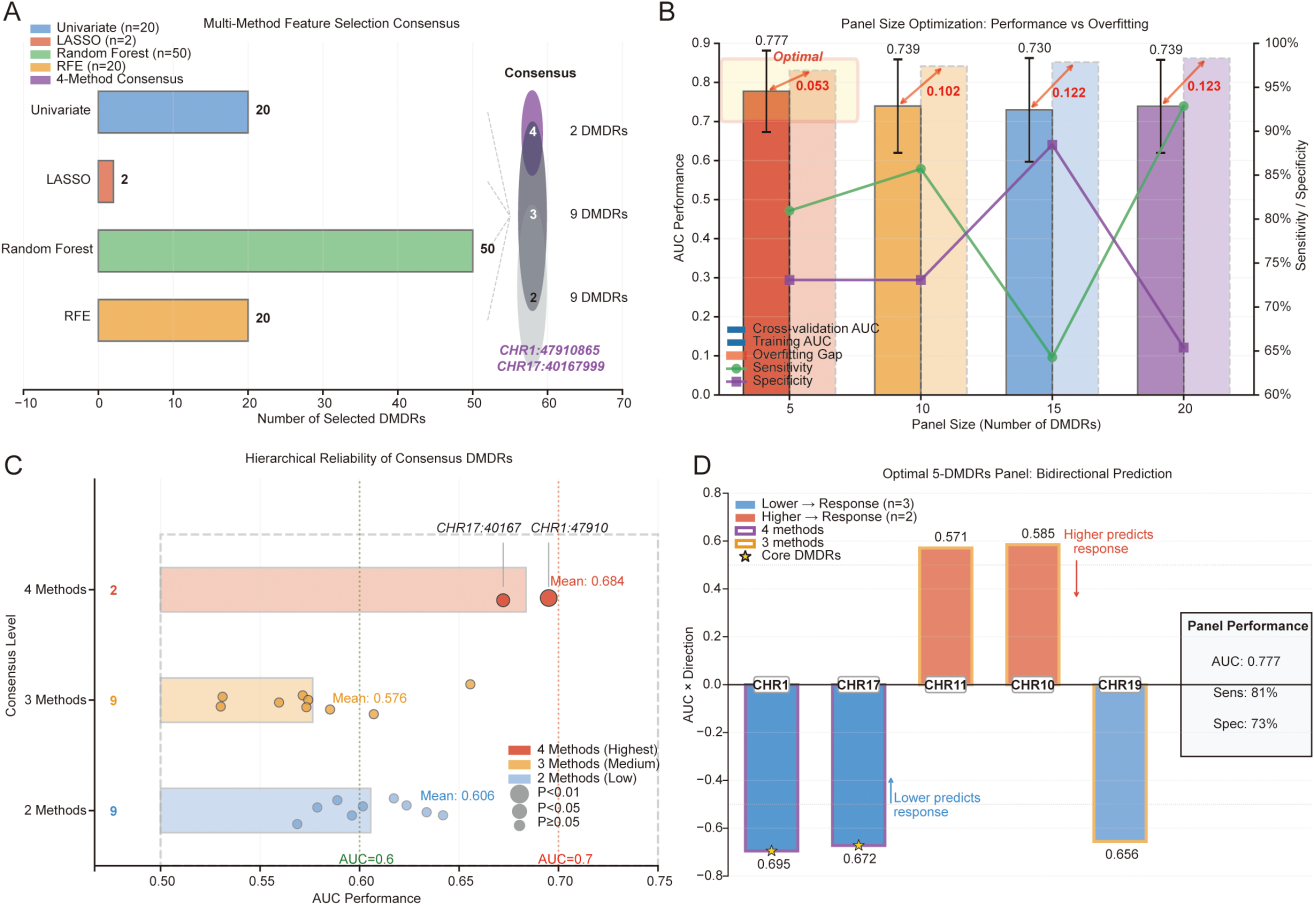
Multi-method feature selection strategy and optimal panel construction. **(A)** Multi-method feature selection consensus analysis. Bar lengths represent DMDR counts selected by each method. Circles show consensus results: purple (4 methods), dark gray (3 methods), light gray (2 methods). **(B)** Panel size optimization analysis. Dark bars represent cross-validation AUC, light bars represent training AUC. Red arrows indicate overfitting difference. Yellow background highlights 5-DMDR panel. **(C)** Reliability gradient stratification of consensus DMDRs. Dot size indicates significance level. Green and red dashed lines mark AUC = 0.6 and AUC = 0.7 thresholds. **(D)** Bidirectional prediction pattern of optimal 5-DMDR panel. Upward bars (red) indicate “high disorder predicts response”; downward bars (blue) indicate “low disorder predicts response.” Gold asterisks mark core DMDRs selected by all methods.

To identify optimal DMDR combinations, we applied four complementary feature selection methods (**Figure 6A**): Random Forest (50 DMDRs selected), univariate selection (20 DMDRs), recursive feature elimination (20 DMDRs), and LASSO regression (2 DMDRs). Consensus analysis identified only 2 DMDRs selected by all four methods: CHR1:47910865–47912865 and CHR17:40167999-40169999. An additional 9 DMDRs were selected by three methods, and 9 by two methods.

Systematic evaluation of different panel sizes revealed clear advantages for streamlined configurations (**Figure 6B**). The 5-DMDR panel achieved the highest cross-validation AUC (0.777), outperforming 10-DMDR (0.739), 15-DMDR (0.730), and 20-DMDR (0.739) panels. Critically, the gap between training and cross-validation AUC widened with panel size: 0.053 for 5-DMDR, 0.102 for 10-DMDR, 0.122 for 15-DMDR, and 0.123 for 20-DMDR, indicating that overfitting risk positively correlated with panel size.

Reliability stratification confirmed this finding (**Figure 6C**): the 2 core DMDRs selected by all four methods showed highest reliability (mean AUC = 0.684), while DMDRs selected by three methods (mean AUC = 0.576) or two methods (mean AUC = 0.606) showed reduced and less consistent performance.

The finalized 5-DMDR panel exhibited a bidirectional prediction pattern (**Figure 6D**). Three DMDRs showed “low disorder predicts response”: CHR1 (AUC = 0.695), CHR17 (AUC = 0.672), and CHR19 (AUC = 0.656). Two additional DMDRs showed “high disorder predicts response”: CHR11 (AUC = 0.571) and CHR10 (AUC = 0.585). This bidirectional pattern suggests that methylation stability in some genomic regions may favor treatment response, while moderate disruption in others may enhance drug sensitivity.

The optimal 5-DMDR panel achieved combined AUC of 0.777 (**Figure 5B**), with 81% sensitivity, 73% specificity, positive predictive value of 81.2%, and minimal overfitting risk (difference of 0.053). The integrated LPMD score distribution showed clear separation between responders and non-responders at the optimal threshold (**Figure 5D**). Bootstrap resampling (1,000 iterations) yielded a 95% confidence interval for the 5-DMDR panel AUC, confirming the stability of this estimate. As a complementary conservative assessment, leave-one-out cross-validation (LOOCV) produced an AUC of 0.621 (accuracy = 55.9%, sensitivity = 66.7%, specificity = 38.5%). While lower than 5-fold CV-–consistent with the known high-variance property of LOOCV in small samples-–this estimate remained above chance (AUC = 0.5), providing a lower-bound confirmation that the predictive signal is genuine rather than an artifact of the cross-validation partitioning scheme.

### Dynamic monitoring during azacitidine treatment

Longitudinal analysis of 153 paired samples across treatment timepoints revealed important temporal patterns (**Figure 5E**). Mean LPMD at diagnosis was 0.665, with only slight decrease (0.6%) at < 3 months of treatment. A significant sharp decrease to 0.640 (-3.7%, *p* < 0.001) occurred at 3–5 months, establishing a critical efficacy assessment window. Thereafter, LPMD levels stabilized at 0.642 (-3.5%) at 6–11 months and 0.643 (-3.2%) at ≥12 months. The Jonckheere-Terpstra trend test confirmed this significant decreasing pattern (Z = -6.87, *p* < 0.0001).

Tracking of five representative DMDRs revealed heterogeneous treatment responses (**Figure 5F**). CHR13:20765 showed the greatest early response with 7.9% decline at 3–5 months, followed by partial rebound to 4.2% below baseline at ≥12 months-–potentially reflecting emergence of resistant clones. CHR17:55331 demonstrated sustained steady decline from ~0.57 at diagnosis to ~0.54 at ≥12 months (cumulative 5.2% decrease). Three regions with high baseline LPMD (CHR7:11696, CHR16:81271, CHR14:10336; values 0.62-0.77) remained relatively stable with only slight decreases (2-3%).

These dynamic patterns have significant clinical implications. First, identification of the 3–5 month critical window provides a key timepoint for treatment efficacy assessment. Second, heterogeneous DMDR responses suggest that treatment strategies may need optimization based on specific genomic regional features. Finally, despite large variation in absolute LPMD values across DMDRs, consistent downward trends support LPMD as a potential biomarker for monitoring azacitidine therapy.

### Convergence of multi-level validation evidence

Taken together, the validation results presented above provide convergent evidence across five complementary dimensions. Internal cross-validation demonstrated that the 5-DMDR panel generalizes beyond the training data (5-fold AUC = 0.777, train-CV gap = 0.053), with LOOCV providing a conservative lower-bound estimate (AUC = 0.621) and bootstrap resampling quantifying parameter uncertainty. Independent cross-platform validation in the WGBS cohort confirmed that LPMD-based differences are not platform-specific artifacts (Cohen’s d = 0.48, 68% directional consistency). Longitudinal treatment dynamics demonstrated that LPMD is not merely a static classifier but dynamically tracks therapeutic response (Jonckheere-Terpstra Z = −6.87, *p* < 0.0001), with the “mutation-associated elevation, treatment-induced reduction” pattern supporting a causal link to the DNMT3A pathway. Finally, multi-method consensus feature selection confirmed that the two core DMDRs (CHR1:47910865-47912865, CHR17:40167999-40169999) were identified by all four independent algorithms, ruling out method-specific bias. The convergence of these distinct analytical approaches-–each addressing a different source of potential error-–collectively supports the robustness and clinical relevance of the LPMD biomarker framework (**Supplementary Table 1**).

## Discussion

This study establishes a local promoter methylation disorder (LPMD) algorithm that fundamentally reframes our understanding of DNMT3A-mutant AML from a “hypomethylation” paradigm to a “methylation disorder” paradigm. Unlike conventional approaches that quantify mean methylation levels, LPMD captures the stochastic nature of epigenetic dysregulation-–a dimension increasingly recognized as central to tumor heterogeneity and therapeutic resistance (23, 33).

The bidirectional disorder pattern we identified-–85.3% of DMDRs showing aberrant stabilization versus 14.7% showing maintenance failure-–reveals an unexpected dichotomy in DNMT3A function. This apparent paradox can be reconciled by considering DNMT3A’s dual roles: as a *de novo* methyltransferase establishing new methylation marks, and as a maintenance factor preserving methylation fidelity at specific loci (34). The R882 hotspot mutation, through its dominant-negative effect on tetramer formation, may simultaneously cause hyperstable methylation in regions dependent on DNMT3A’s catalytic activity and destabilize regions requiring its scaffolding function for recruiting maintenance machinery (7). Notably, the disorder-increased regions are enriched in promoters and CpG islands-–precisely where the DNMT3A-DNMT3L complex normally ensures methylation precision through its interaction with unmethylated H3K4 (35). Loss of this targeting mechanism likely underlies the stochastic methylation we observe.

The concentrated disorder in HOX gene clusters (HOXA7, HOXB4, HOXB5, HOXB6, HOXC8; d = 0.58-0.92) has profound mechanistic implications. DNMT3A directly regulates methylation at HOX gene loci in hematopoietic stem cells, and conditional Dnmt3a knockout leads to aberrant HOX gene activation and impaired differentiation (49). HOX genes are organized in topologically associated domains (TADs) where methylation states propagate across regulatory elements, and methylation disorder at these loci disrupts the coordinated silencing-activation transitions essential for hematopoietic lineage commitment (36). Importantly, Lu et al. demonstrated that DNMT3A-R882H directly binds to and potentiates transactivation of stemness genes including Meis1 and the Hoxa cluster, inducing focal CpG hypomethylation with concurrent gain of H3K4me3 at cis-regulatory enhancers (51). The HOXA9/MEIS1 transcriptional axis is one of the most potent oncogenic drivers in AML, overexpressed in over 70% of cases, and our observation that multiple HOX family members simultaneously exhibit increased LPMD suggests that R882-driven methylation disorder destabilizes the entire HOX regulatory network rather than affecting individual genes in isolation. This positions HOX loci as potential therapeutic targets for epigenetic intervention.

The identification of PRDM16 as the central network hub-–recurring in 15 independent DMDRs-–provides additional mechanistic depth. PRDM16 (also known as MEL1) is a physiologic regulator of hematopoietic stem cells expressed selectively in the earliest stem and progenitor compartments, where it maintains HSC quiescence and integrates self-renewal, cycling, and apoptosis (50). Its translocation t(1;3)(p36;q21) is a recurrent event in MDS/AML. Critically, PRDM16 functions as an intrinsic histone methyltransferase that suppresses leukemogenesis by directly activating GFI1b, which in turn downregulates the HOXA gene cluster; silencing of PRDM16 by DNA methylation accompanies leukemic transformation (52). Thus, methylation disorder at PRDM16-regulated loci may simultaneously de-repress HOX genes and compromise the balance between self-renewal and differentiation that is central to leukemogenesis. The modular network organization-–with PRDM16 connecting a developmental regulation module (HOX genes) and a metabolic regulation module-–suggests that DNMT3A mutations disrupt coordinated biological programs rather than random genomic regions.

Consistent with this gene-level evidence, pathway enrichment analysis revealed that genes with elevated LPMD are significantly enriched in DNA binding and transcription regulation functions (FDR < 0.05), with nominal enrichment in the AML disease pathway (FLT3, RUNX1, ZBTB16) and hematopoietic progenitor differentiation (SHH, FLT3, HOXB4). Among individual genes, MSI2-–a Musashi family RNA-binding protein that regulates HSC self-renewal through the Numb/Notch axis and whose overexpression promotes aggressive myeloid leukemia (53)-–and WNT2 ranked among our top DMDRs with the largest effect sizes (d = 1.21 and 1.26, respectively), further underscoring the biological coherence of LPMD-affected loci with known leukemogenic pathways. This convergence on transcription regulatory networks aligns with DNMT3A’s known role as a regulator of gene expression programs. Recent single-cell multi-omic profiling has demonstrated that R882 mutations cause selective hypomethylation at Polycomb Repressive Complex 2 (PRC2) targets and specific CpG flanking motifs rather than random genome-wide demethylation (54), explaining why LPMD changes concentrate at developmental regulatory loci. R882 dominant-negative disruption of DNMT3A tetramerization preferentially destabilizes methylation at regulatory elements where precise epigenetic control is required, while sparing repetitive and structural regions that rely on maintenance methylation (8). The resulting model is one of targeted regulatory chaos-–DNMT3A mutations selectively disorder the epigenetic control of master transcription factor networks, propagating downstream effects through developmental signaling cascades.

A counterintuitive finding emerged from our treatment response analysis: genome-wide LPMD failed to predict azacitidine response, yet region-specific DMDRs achieved robust prediction (AUC = 0.777). This dissociation suggests that therapeutic vulnerability resides not in global epigenetic chaos but in the disorder status of specific regulatory nodes. The “low disorder predicts response” pattern implies that patients retaining epigenetic plasticity-–the capacity for ordered methylation remodeling-–respond better to demethylating agents. Conversely, highly disordered epigenomes may have already undergone irreversible “epigenetic drift” beyond therapeutic rescue (37, 38). This concept parallels the “epigenetic age” hypothesis, where accumulated stochastic methylation changes correlate with biological dysfunction (39).

The temporal dynamics of LPMD during treatment provide mechanistic insights into azacitidine action. The pronounced decrease at 3–5 months (-3.7%, *p* < 0.001) followed by stabilization suggests a biphasic response: initial elimination of disordered clones, followed by equilibrium between drug-induced demethylation and compensatory remethylation. The partial rebound observed in specific DMDRs (e.g., CHR13 region) may herald the emergence of resistant subclones with adapted methylation machinery (40). This dynamic signature could serve as an early indicator for therapeutic switching before clinical relapse manifests.

The superiority of the minimalist 5-DMDR panel over larger configurations reflects a fundamental principle in biomarker development: biological signal concentrates in key regulatory nodes while noise accumulates with feature expansion (41). The bidirectional composition of this panel-–three “low disorder predicts response” and two “high disorder predicts response” DMDRs-–captures the mechanistic complexity of treatment response and may reflect distinct pathways: direct cytotoxicity in ordered regions versus differentiation induction in disordered regions (42).

Importantly, the five-layer complementary validation framework employed in this study-–spanning internal cross-validation, bootstrap robustness assessment, independent cross-platform validation, longitudinal dynamic tracking, and multi-method consensus feature selection-–provides convergent evidence from distinct analytical dimensions, supporting the reliability and clinical applicability of the LPMD approach despite the limitations discussed below.

Several limitations warrant consideration. The discovery cohort size (n = 15 DNMT3A-mutant vs. n = 53 wild-type) limits statistical power for detecting small effect sizes. *Post-hoc* power analysis based on the non-central t-distribution showed that the minimum detectable effect size at 80% power was Cohen’s d = 0.83 for our sample configuration (α = 0.05, two-sided). All Tier 1 and Tier 2 DMDRs (|d| > 1.0, n = 16) achieved greater than 99% power, confirming robust detection of the strongest signals. However, the mean effect size across all 7,097 DMDRs (|d| = 0.675) corresponded to approximately 62% power, indicating that Tier 3 and Tier 4 DMDRs with moderate effect sizes may have reduced detection sensitivity-–consistent with their exploratory classification. Gene-level conclusions should therefore be considered exploratory and require validation in larger cohorts. The consistent direction of effects in the independent WGBS validation cohort nonetheless supports the overall biological validity of our findings. The validation cohort, though demonstrating directional consistency (68% of DMDRs), showed attenuated effect sizes, reflecting biological heterogeneity across patient populations and technical differences between platforms. Additionally, neither the discovery cohort (GSE62298) nor the WGBS validation cohort provide R882 subtype annotations. To address this limitation, we performed R882-stratified LPMD analysis in the TCGA-LAML cohort (n = 194), where codon-level DNMT3A mutation data were available (R882: n = 27; non-R882: n = 21; wild-type: n = 146). R882 mutants exhibited significantly elevated LPMD compared to wild-type (Mann-Whitney *p* = 7.8 × 10^-5^, Cohen’s *d* = 0.577), whereas non-R882 mutations showed a non-significant intermediate elevation (*p* = 0.30), consistent with the heterogeneous functional impact of non-R882 variants. Gene-level analysis identified 4,148 genes with significantly differential LPMD (FDR < 0.05), with 70% showing higher disorder in R882 mutants (**Supplementary Tables 4**, **S5**). These results confirm that the dominant-negative R882 mechanism produces a stronger and more uniform LPMD signature, while the LPMD algorithm itself captures methylation coordination loss regardless of the specific upstream mutation mechanism. The mechanism underlying region-specific bidirectional disorder remains incompletely characterized and likely involves interactions with co-mutated genes (TET2, IDH1/2) that modulate the epigenetic landscape (43). Integration with chromatin accessibility and three-dimensional genome organization data will be essential to fully resolve the regulatory consequences of methylation disorder (44).

In conclusion, the LPMD algorithm provides a new lens for understanding DNMT3A-mutant AML-–one that emphasizes methylation stability rather than level. The practical 5-DMDR panel offers a clinically implementable tool for azacitidine response prediction, while dynamic LPMD monitoring opens possibilities for real-time therapeutic guidance. Future prospective trials integrating LPMD with mutational profiling and gene expression signatures may enable truly personalized epigenetic therapy in myeloid malignancies.

## Data Availability

The datasets presented in this study can be found in online repositories. The names of the repository/repositories and accession number(s) can be found in the article/**Supplementary Material**.
