# Supplementary figures and images for: Local Promoter Methylation Disorder algorithm reveals bidirectional epigenetic disruption in DNMT3A-mutated AML and predicts azacitidine treatment response
Source: Front Oncol. 2026 Mar 18;16:1795605. doi: 10.3389/fonc.2026.1795605 (PMC13038435; doi:10.3389/fonc.2026.1795605)

Top GO Terms

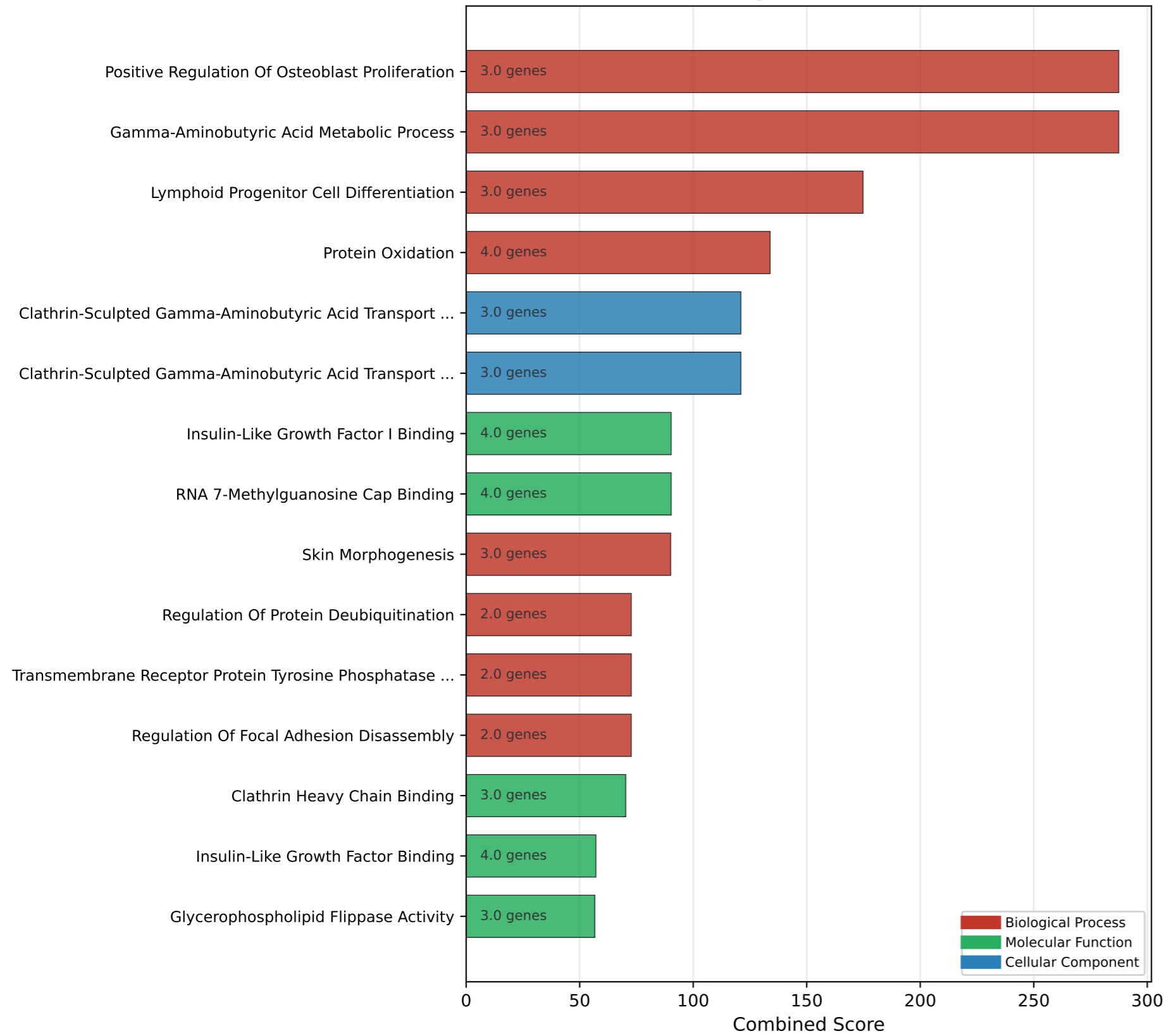

Top KEGG Pathways

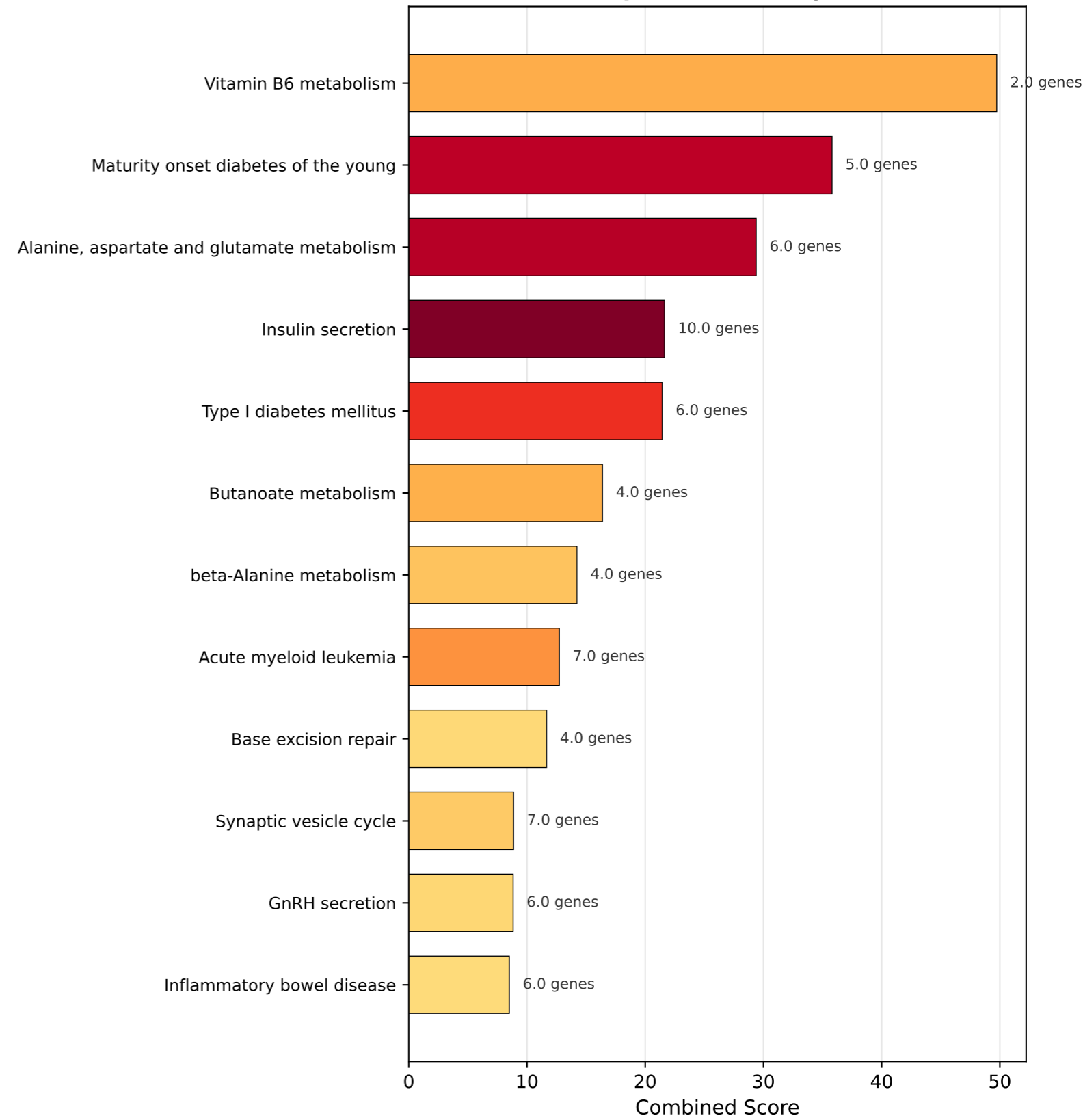

Supplement: Supplementary file 1 [file DataSheet1.pdf]

**(A)**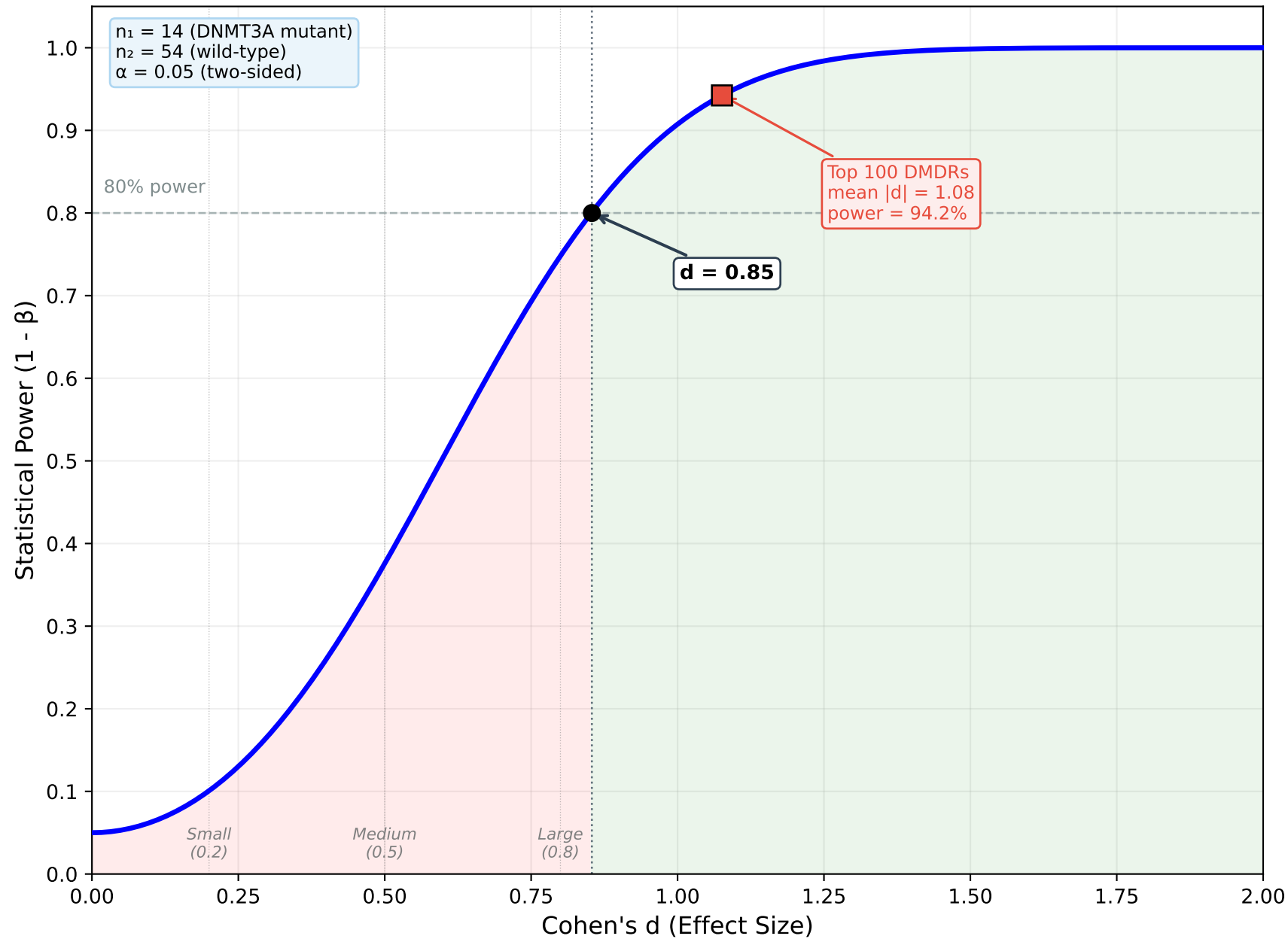**(B)**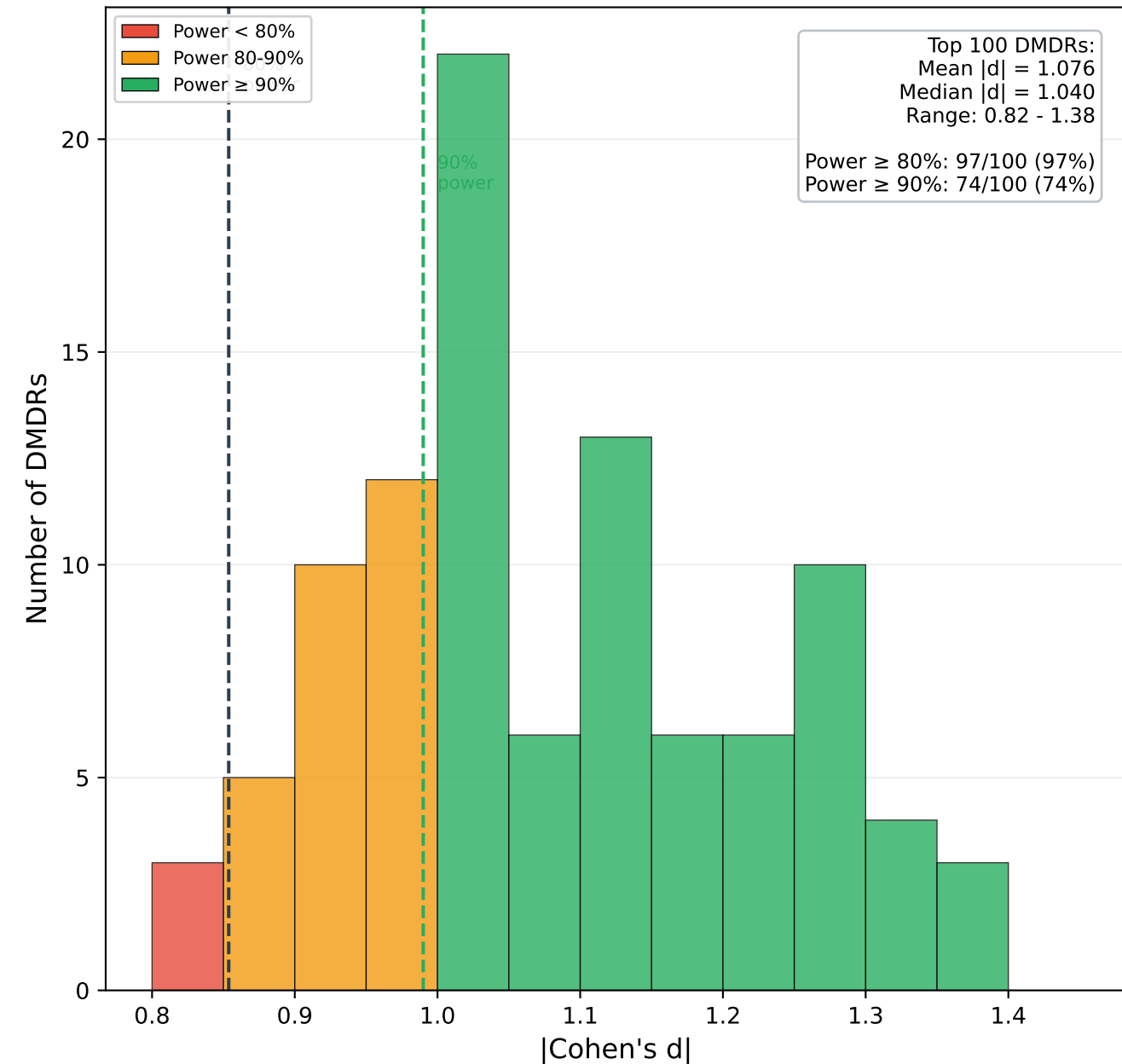

Supplement: Supplementary file 2 [file DataSheet2.pdf]
